# Corticosteroids pulse therapy and oral corticosteroids therapy for IgA nephropathy patients with advanced chronic kidney disease: results of a multicenter, large-scale, long-term observational cohort study

**DOI:** 10.1186/s12882-018-1019-x

**Published:** 2018-09-06

**Authors:** Ryoya Tsunoda, Joichi Usui, Junichi Hoshino, Takayuki Fujii, Satoshi Suzuki, Kenmei Takaichi, Yoshifumi Ubara, Kunihiro Yamagata

**Affiliations:** 1 Department of Nephrology, Faculty of Medicine, University of Tsukuba, 1-1-1 Tennodai, Tsukuba, Ibaraki, 305-8575 Japan; 2 Nephrology Center, Toranomon Hospital, Tokyo, Japan; 3 Department of Nephrology, Seirei Sakura Citizen Hospital, Sakura, Japan; 4 Nephrology Center, Toranomon Hospital Kajigaya, Kawasaki, Japan

**Keywords:** IgA nephropathy, Glomerulonephritis, Chronic kidney disease, Corticosteroids pulse therapy

## Abstract

**Background:**

Corticosteroids are widely used to reduce the urine protein levels of patients with immunoglobulin A nephropathy (IgAN). However, their potential preventive effects on end-stage kidney disease (ESKD) are unclear.

**Methods:**

We previously performed a large-scale, long-term multicenter cohort study of patients with biopsy-proven IgAN treated between 1981 and 2013 (*n* = 1923). Based on the results, we reported that corticosteroids pulse therapy was potentially effective for the treatment of patients with an eGFR ≥30 ml/min/1.73m^2^ and a urine protein amount of ≥1 g/gCr. In the present study, we extracted 766 patients with chronic kidney disease (CKD), stage G3–G4 (15 ≤ estimated glomerular filtration rate [eGFR] < 60 mL/min/1.73m^2^) from the same cohort. We divided these patients into a steroid pulse (SP) group, oral steroid (OS) group, and no steroid (NS) group, and analyzed the risk of end-stage kidney disease (ESKD) stratified by eGFR and urine protein (UP) amounts.

**Results:**

Over the median long-term follow-up of 70 ± 115 months, 37.1% of the patients with UP ≥1.0 g/day and 11.2% of the patients with UP < 1.0 g/day reached ESKD. Among the patients with UP ≥1 g/gCr, the SP group showed significantly better renal outcome (*p* < 0.001) than the OS and NS groups. In patients with UP < 1 g/gCr, there were no differences in renal survival among the treatment groups. These trends appeared even in the CKD stage G4 patients, and were also apparent in patients taking renin-angiotensin system inhibitors. The unprecedented long-term observation period in this study may have been necessary to reveal the favorable effect of corticosteroids on ESKD progression.

**Conclusions:**

In our long-term multicenter study, Corticosteroids pulse therapy was associated with better renal outcomes in IgAN patients with higher UP values, even if their eGFR values were low.

## Background

Immunoglobulin A nephropathy (IgAN), a form of glomerulonephritis characterized by the mesangial deposition of immunoglobulin A (IgA), is the most common cause of chronic nephritis around the world [1]. Since the 1980s, corticosteroid administration has been used worldwide as a treatment for IgAN, and many studies (including several controlled trials) have analyzed the efficacy of corticosteroids for this purpose [2–11]. Almost all these studies reported that corticosteroids therapy reduced the urine protein level, which is a surrogate marker for progression of renal impairment. However, there is little evidence of an improvement in renal survival itself. Thus the indications, optimal treatment method, and effectiveness of corticosteroids administration for IgAN are unclear, and controversy remains about this treatment approach.

In 1999, Pozzi et al. reported a randomized controlled trial (RCT) of Corticosteroids pulse treatment for IgAN patients with urine protein levels of 1.0–3.5 g/day [6, 12], with 3 days of intravenous corticosteroids administration in months 1, 3, and 5. They reported that corticosteroids ameliorated the decline in renal function of patients during the mean follow-up period of 5 years, and the results of their RCT have provided important evidence supporting the effectiveness of Corticosteroids pulse treatment for patients with IgAN.

The results of more recent studies are quite complicated. In 2015, Rausen et al. reported the results of the Stop-IgAN RCT of immunosuppression treatment for IgAN [13]. They stated that the administration of immunosuppressive drugs including corticosteroids in addition to intensive supportive care including renin-angiotensin inhibition did not improve the decrease in the estimated glomerular filtration rate (eGFR) in a 3-year follow-up. Several months after the Stop-IgAN trial, Tesar et al. reported the European Validation Study of the Oxford Classification of IgAN (VALIGA), noting that corticosteroids tended to improve renal outcomes in a 10-year follow-up [14]. Several similar studies excluded patients with reduced renal function.

In many cases of IgA nephropathy, worsening of renal function takes a long time. Accordingly, a long-term cohort study may be the only way to reveal renal outcome, especially for serious outcomes such as end-stage kidney disease (ESKD). Therefore, we previously established a large-scale and long-term multicenter cohort study of patients with biopsy-proven IgAN, with a long-term median follow-up duration of 70.0 months [15]. Based on the results of that study, we reported that steroid pulse therapy with or without tonsillectomy was potentially effective for IgAN patients with chronic kidney disease (CKD) stage G1–G3 and high urine protein levels [15]. Our analyses indicated that tonsillectomy did not seem to enhance the benefit of corticosteroids monotherapy. We thus considered that it would be important to estimate the effectiveness of corticosteroids when used alone.

However, the effectiveness of corticosteroids treatment for IgAN patients with reduced renal function has remained controversial. In the present study, therefore, we examined the renal prognosis of patients with advanced CKD from our previously established large-scale cohort.

## Methods

### Patient recruitment

We previously reported a multicenter retrospective cohort study of biopsy-proven IgAN patients at four kidney centers in Japan: the University of Tsukuba, Toranomon Hospital, Seirei Sakura Citizen Hospital, and Toranomon Hospital Kajigaya [15]. The 1923 patients who underwent a kidney biopsy at one of these centers during the period from March 1, 1981 to December 31, 2013 were recruited. Each patient’s observation began, and all baseline parameters were recorded, at the time of the biopsy. The study’s clinical endpoint was end-stage kidney disease (ESKD), which was defined as the initiation of renal replacement therapy. Renal replacement therapy, in turn, was defined as dialysis or pre-emptive kidney transplantation, though no patients underwent transplantation during our observation. Dialysis was initiated by a physician’s decision in each patient, in accordance with the criteria generally used for dialysis initiation in Japan, i.e., serum creatinine of ≥8 mg/dl, uremic symptoms, or lowered ADL due to chronic renal failure. This practice did not vary over the approximately 30-year study period. All patients were observed until ESKD, death, or censoring. The mean follow-up period of the whole cohort was 8.3 years.

We categorized the patients into three groups based on the method of corticosteroids administration. The steroid pulse (SP) group included patients who received intravenous methylprednisolone (mPSL) pulse therapy for at least 3 days in a row during their follow-up period. Typical steroid pulse therapy in this study was administered over a 3-week period, with a single course of methylprednisolone (500 mg/day) for 3 days followed by oral corticosteroids, according to the protocol reported by Hotta et al. [7]. However, each patient’s protocol could be altered by the treating physician according to the patient’s status, since at the time of our study there was currently no firm evidence or established regimen to guide the administration of corticosteroids in patients with IgA nephropathy. Thus, a single dosage of methylprednisolone varied from 500 mg to 1000 mg, the number of doses varied from one to three courses in a single treatment series, and the tapering schedules for the corticosteroids also varied.

The oral steroid (OS) group included the patients who received corticosteroids without an intravenous pulse. The no steroid (NS) group did not receive any corticosteroids. In light of our previous finding [15], we did not investigate the influence of tonsillectomy.

From the 1923 patients in the total cohort, we extracted the cases of the 764 patients with chronic kidney disease (CKD), stage G3–G4 (15 ≤ eGFR < 60 mL/min/1.73m^2^) as subjects for the present study. The eGFR was calculated by the formula for Japanese created by Matsuo et al. [16].

### Statistical analyses

We stratified the cases by the amount of urine protein (UP), which was calculated as the urine protein to urine creatinine ratio (g/gCr), and investigated the renal survival. The data are summarized as proportions (percent), median (1st interquartile – 3rd interquartile) or means (± standard deviation). Categorical variables were analyzed by Fisher’s exact test, and continuous variables by Student’s t-test. The survival analysis was performed by the Kaplan-Meier method and with the log-rank test. Hazard ratios of ESKD were calculated according to a Cox-proportional hazard model. All analyses were conducted using the R software program, ver. 3.4.2 (The R Foundation for Statistical Computing Platform: x86_64-w64-mingw32/× 64 (64-bit)).

## Results

### The subjects’ baseline characteristics

Table 1 summarizes the baseline characteristics of the 764 subjects (319 females, 445 males). The mean age was 50.3 ± 13.6 years. The median follow-up period was 70 months. The median UP at the time of the biopsy was 0.95 g/gCr. The numbers of patients with CKD stages G3a (45 ≤ eGFR < 60 mL/min/1.73m^2^), G3b (30 ≤ eGFR < 45 mL/min/1.73m^2^), and G4 (15 ≤ eGFR < 30 mL/min/1.73m^2^) were 397 (52.0%), 236 (30.9%), and 131 (17.1%), respectively.

**Table 1:** Baseline characteristics of the subjects at the time of the renal biopsy

Characteristic	Whole G3–4	CKD G stage				Treatment		
		G3a	G3b	G4		Pulse (SP)	Oral (OS)	None (NS)
*n*	764	397	236	131		121	139	506
Age, years	50.3 ± 13.6	47.7 ± 13.8	52.3 ± 13.4	54.6 ± 12.8		49.3 ± 14.7	49.5 ± 13.2	50.8 ± 13.4
Gender								
Female	319 (42)	171 (57)	90 (38)	58 (44)		45 (37)	53 (38)	221 (44)
Male	445 (58)	226 (43)	146 (62)	73 (56)		76 (63)	86 (62)	283 (56)
Follow-up, months	70 ± 115	86 ± 141	65 ± 103	44 ± 61		67 ± 81	88 ± 125	65 ± 121
eGFR, ml/min/1.73m^2^	43 ± 12	53 ± 4.2	38 ± 5.6	23 ± 4.5		41 ± 13	41 ± 13	44 ± 11
CKD G Stage								
G3a	397 (52)	397 (100)	–	–		56 (46)	64 (46)	277 (55)
G3b	236 (31)	–	236 (100)	–		38 (31)	45 (32)	153 (30)
G4	131 (17)	–	–	131 (100)		27 (22)	30 (22)	74 (15)
UP, g/gCr	0.95 ± 1.4	0.70 ± 1.1	1.2 ± 1.7	1.8 ± 2.7		1.5 ± 1.9	1.8 ± 2.2	0.73 ± 1.1
< 1.0	385 (50)	248 (62)	94 (40)	43 (33)		47 (39)	45 (32)	309 (61)
≥ 1.0	363 (48)	141 (36)	136 (58)	86 (66)		74 (61)	94 (68)	195 (39)
sBP, mmHg	134 ± 19	129 ± 19	137 ± 20	140 ± 19		134 ± 17	135 ± 19	133 ± 20
dBP, mmHg	80 ± 13	78 ± 12	82 ± 13	83 ± 14		79 ± 12	80 ± 13	80 ± 13
RAAs inhibition								
+	209 (27)	104 (26)	70 (30)	34 (26)		62 (51)	55 (40)	92 (18)
-	313 (41)	173 (44)	85 (36)	55 (42)		59 (49)	83 (60)	172 (34
Corticosteroids								
Pulse (SP)	121 (16)	56 (14)	38 (16)	27 (21)		121 (100)	–	–
Oral (OS)	139 (18)	64 (16)	45 (19)	30 (23)		–	139 (100)	–
None (NS)	506 (66)	277 (70)	153 (65)	74 (56)		–	–	506 (100)
Immunosuppressant								
+	10 (2)	4 (1)	5 (2)	1	(1)	4 (3)	6 (4)	0 (0)
-	513 (70)	274 (69)	151 (64)	88	(67)	117 (97)	132 (95)	264 (52)
NA	241 (28)	119 (30)	80 (34)	42	(32)	0 (0)	1 (1)	240 (48)
Tonsillectomy	80 (10)	46 (12)	26 (11)	8	(6)	68 (56)	4 (3)	8 (2)

*CKD* chronic kidney disease, *eGFR* estimated glomerular filtration rate, *immunosuppressive drugs* use of immunosuppressive drugs other than corticosteroids, *NA* not available, *None* no corticosteroids were administered during the observation period, *Oral* corticosteroids were administrated only orally, *Pulse* corticosteroid-pulse therapy was administered during the observation period, *RAAs inhibition* renin-angiotensin-aldosterone system inhibition (use of angiotensin-converting enzyme inhibitors, angiotensin II receptor blockers, or mineralocorticoid blockers), *UP* urine protein to urine creatinine ratio (g/gCr)

Higher ages, shorter follow-up periods, and greater UP values were observed in the further-progressed G stages. The proportions of patients administered corticosteroids by each method were as follows. For the total subject group (G3a, G3b and G4 patients), 16% (*n* = 121), 18% (*n* = 139) and 66% (*n* = 504) of patients received SP, OS and NS treatment, respectively. Among G3a patients, the corresponding percentages were 14% (*n* = 56), 16% (*n* = 64) and 70% (*n* = 277). In G3b patients, they were 16% (*n* = 38), 19% (*n* = 45) and 65% (*n* = 153). And in G4 patients, 21% (n = 27), 23% (*n* = 30) and 56% (*n* = 74) of patients received SP, OS and NS therapy. Tonsillectomy was performed in 80 (10%) patients. Most of them (*n* = 68) were treated by a combination with SP. Only 8 patients underwent tonsillectomy without steroid administration.

Approximately 27% of patients had received RAAS inhibitors. Only 1.6% of subjects received immunosuppressive drugs other than corticosteroids.

### Renal survival

**Fig. 1:**
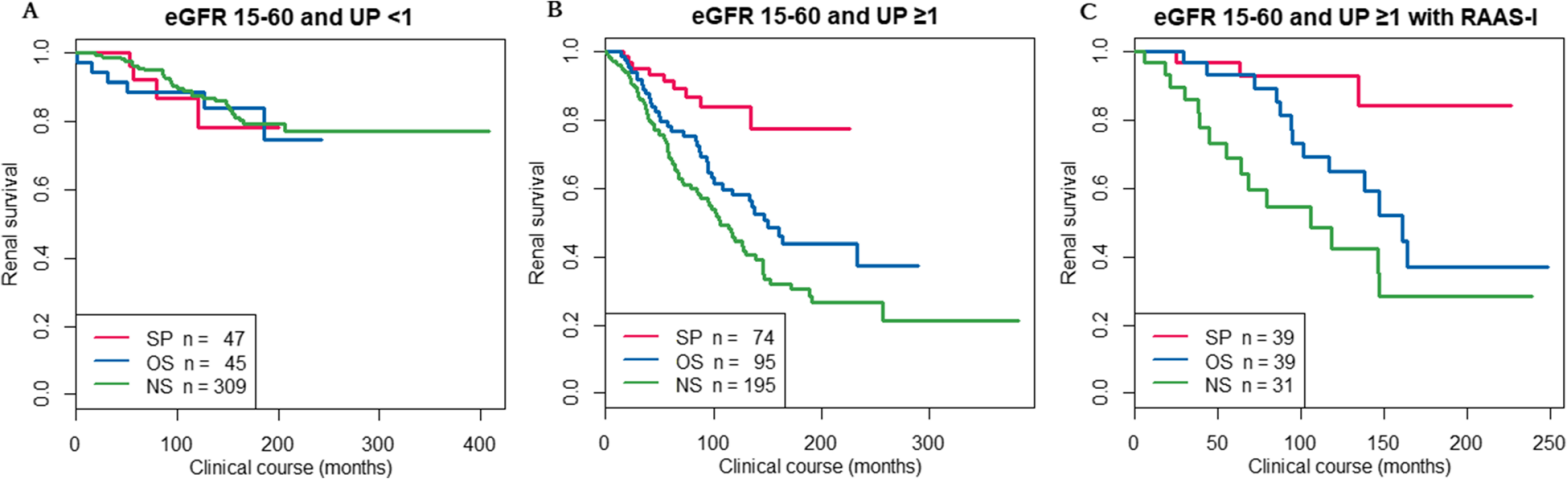
Kaplan-Meier curves of renal survival in the CKD G3–4 patients. A: Patients with UP < 1 g/gCr. B: Patients with UP ≥1 g/gCr. C: patients with UP ≥1 g/gCr and taking an angiotensin converting enzyme inhibitor (ACEi) or angiotensin II receptor blocker (ARB)

During the follow-up period, 37.1% of the patients with UP ≥1.0 g/day and 11.2% of the patients with UP < 1.0 g/day reached ESKD. Figure 1a and b show the Kaplan-Meier curves of renal survival in the patients with G3a, G3b and G4 CKD for each level of UP. Among the patients with UP ≥1 g/gCr, the SP group showed significantly better renal outcomes (*p* < 0.001) compared to the CS and NS groups. Figure 1c shows the Kaplan-Meier curves of renal survival in the patients with G3a, G3b and G4 CKD with UP ≥1 g/gCr and taking renin-angiotensin system inhibitors. Even in this subgroup, SP showed significantly better renal outcome. On the other hand, in patients with UP < 1 g/gCr, there were no significant differences in renal survival among the three treatment groups.

## Discussion

Previous studies of corticosteroids administration for IgAN have reported several negative results, but there are few long-term studies of this treatment. In typical cases of IgA nephropathy, progression of ESKD can take one or more decades. So, as a matter of course, only rare cases reach ESKD within three-years of follow-up, as described in the previous report from our institute [5] or the STOP-IgAN report [13]. Therefore, a long-term observation period like that of the present study might have been necessary to reveal the favorable effects of corticosteroids administration in patients with IgAN. In our study, the favorable therapeutic effects of corticosteroids administration were apparent even in patients with moderate UP (0.5–1 g/gCr) and advanced CKD (stage G4); however, the number of these patients was small (*n* = 23).

There have been few investigations into the efficacy of corticosteroids for IgAN patients with advanced CKD, because patients with reduced eGFR or creatinine clearance (Ccr) have been excluded from most of the prospective studies [6, 7, 9]. In 1986, Kobayashi et al. reported a cohort of IgAN cases with urine protein ≥1 g/day and compared the annual decline of Ccr among patients categorized by their initial Ccr [2–4]. In that study, in 14 patients with Ccr < 70 ml/min, the corticosteroids treatment did not improve renal outcome at a 10-year follow-up; however, the number of patients was insufficient to reach any definitive conclusions. Moreover, their patients received corticosteroids by oral administration, so these findings do not contradict our present results.

Moriyama et al. reported that, among a group of 60 patients with Ccr < 70 mL/min, higher urinary protein, and active glomerular lesions, oral steroid treatment was effective in 20 patients. Lv et al. reported that oral mPSL (0.6–0.8 mg/kg/day) conferred an increased risk of serious adverse events in their IgAN patients, despite the potentially favorable renal outcomes [17]. Although in VALIGA the effectiveness of corticosteroids seemed apparent even in patients with eGFR ≤50 ml/min/1.73m^2^ [14], the method of corticosteroids administration was not analyzed. Our present study may thus be the first report detailing the potential of steroid pulse treatment for IgAN with reduced renal function.

There are several limitations of our study to be considered. First, bias from potential differences among the treatment groups should be mentioned. For example, avoidance of corticosteroids treatment might be associated with comorbidities such as infection, diabetes, cardiovascular disease or other physical conditions that could affect CKD progression. Second, the speed of the reduction of eGFR at the time of the biopsy was not recorded, and we thus could not detect rapidly progressive cases that might be responsive to corticosteroids. Patients with an advanced CKD G-stage in our cohort might have included rapidly progressive cases that would not generally be considered “chronic”. Third, we did not collect data about adverse events. In the future, it will important to evaluate the risk of steroid pulse treatment, which is considered safer than oral corticosteroids therapy [18].

Finally, the fact that few patients received RAAS inhibitors might constitute a major limitation of this study. In 2003, Praga et al. reported the effectiveness of ACE inhibitors for IgAN patients with UP ≥0.5 g/gCr [19]. Since then, RAAS inhibitors have become a worldwide standard therapy for IgAN. Our present cohort included patients treated in the 1980s–2000s, so the patient characteristics may have differed from that of patients in the 2010s. Moreover, in Japan, RAAS inhibitors are not approved as medications for glomerulonephritis, but only for treatment for hypertension or diabetic kidney disease. So it was difficult for us to use RAAS inhibitors for non-diabetic patients with normal blood pressure. Recently, following publication of the KDIGO guidelines, the use of RAAS inhibitors has been increasing in Japan. Though the benefit of pulse therapy was apparent even if we extracted patients who received RAAS inhibitors, this might be a limitation for interpreting our results.

In summary, among our study’s patients with UP ≥1 g/gCr, those who were treated with steroid pulse (SP) therapy showed the best renal outcomes. In light of this result, Corticosteroids pulse treatment was associated with better renal outcomes in IgAN patients with higher UP values, even if their eGFR values are low. A high urine protein value might indicate disease with high activity (which is responsive to immunosuppression) in contrast to stable CKD with low UP values.

## Conclusions

We showed the potential favorable effect of Corticosteroids pulse therapy on IgAN patients with reduced renal function. We need to create a unified regimen of Corticosteroids pulse therapy for IgAN in order to identify predictors for corticosteroids sensitivity, and we must determine the precise effectiveness and safety of this therapy.

## Abbreviations

Ccr: Creatinine clearance
CKD: Chronic kidney disease
eGFR: Estimated glomerular filtration rate
ESKD: End-stage kidney disease
IgA: Immunoglobulin A
IgAN: Immunoglobulin A Nephropathy
IRB: Institutional review board
mPSL: Methylprednisolone
NA: Not available
NS: No steroid
OS: Oral steroid
RAAs: Renin-angiotensin-aldosterone system
RCT: Randomized controlled trial
SP: Steroid pulse
UP: Urine protein
